# Gamma-type immunoglobulin enhances phagocytosis of amyloid-beta fibrils by microglia

**DOI:** 10.4103/NRR.NRR-D-23-01942

**Published:** 2024-07-29

**Authors:** Tian Zhou, Yue Zhong, Shoujun Yu, Ruibing Sun, Zhenwei Zhang, Xiaoyan Du, Simon Ming-Yuen Lee, Zhitong Chen, Weiming Tian, Yuxiao Lai, Bing Song, Yiming Zheng, Zhen Xu

**Affiliations:** 1 Key Laboratory of Mental Health of the Ministry of Education, Guangdong-Hong Kong-Macao Greater Bay Area Center for Brain Science and Brain-Inspired Intelligence, Guangdong-Hong Kong Joint Laboratory for Psychiatric Disorders, Guangdong Province Key Laboratory of Psychiatric Disorders, Guangdong Basic Research Center of Excellence for Integrated Traditional and Western Medicine for Qingzhi Diseases, Department of Neurobiology, School of Basic Medical Sciences, Southern Medical University, Guangzhou, Guangdong Province, China; 2 Shenzhen Institutes of Advanced Technology, Chinese Academy of Sciences, Shenzhen, Guangdong Province, China; 3 Shenzhen Hospital, Southern Medical University, Shenzhen, Guangdong Province, China; 4 Department of Dermatology, The First Hospital of China Medical University, Shenyang, Liaoning Province, China; 5 Department of Ophthalmology, The Seventh Affiliated Hospital, Sun Yat-sen University, Shenzhen, Guangdong Province, China; 6 State Key Laboratory of Cellular Stress Biology, School of Life Sciences, Faculty of Medicine and Life Sciences, Department of Neurology, Xiang’an Hospital of Xiamen University, Xiamen University, Xiamen, Fujian Province, China; 7 Shenzhen Research Institute of Xiamen University, Shenzhen, Guangdong Province, China; 8 Department of Food Science and Nutrition, The Hong Kong Polytechnic University, Hong Kong Special Administration Region, China; 9 National Innovation Center for Advanced Medical Devices, Shenzhen, Guangdong Province, China; 10 School of Life Science and Technology, Harbin Institute of Technology, Harbin, Heilongjiang Province, China

**Keywords:** adaptive immunity, Alzheimer’s disease, amyloid-beta, B cell, blood–brain barrier, cell membrane, Fc fragment, gamma-type immunoglobulin, microglia, phagocytosis

## Abstract

The peripheral immune system has emerged as a regulator of neurodegenerative diseases such as Alzheimer’s disease. Microglia are resident immune cells in the brain that may orchestrate communication between the central nervous system and peripheral immune system, though the mechanisms are unclear. Here, we found that gamma-type immunoglobulin, a product originating from peripheral blood B cells, localized in the brain parenchyma of multiple mouse models with amyloid pathology, and was enriched on microglia but not on other brain cell types. Further experiments showed that gamma-type immunoglobulin bound to microglial cell membranes and led to diverse transcriptomic changes, including upregulation of pathways related to phagocytosis and immunity. Functional assays demonstrated that gamma-type immunoglobulin enhanced microglial phagocytic capacity for amyloid-beta fibrils via its Fc fragment, but not Fab fragment, fragment. Our data indicate that microglia, when exposed to gamma-type immunoglobulin, exhibit an enhanced capacity for clearing amyloid-beta fibrils, potentially via the gamma-type immunoglobulin Fc fragment signaling pathway. This suggests that parenchymal gamma-type immunoglobulin should be further investigated to determine whether it may play a beneficial role against Alzheimer’s disease by enhancing microglial function.

## Graphical abstract

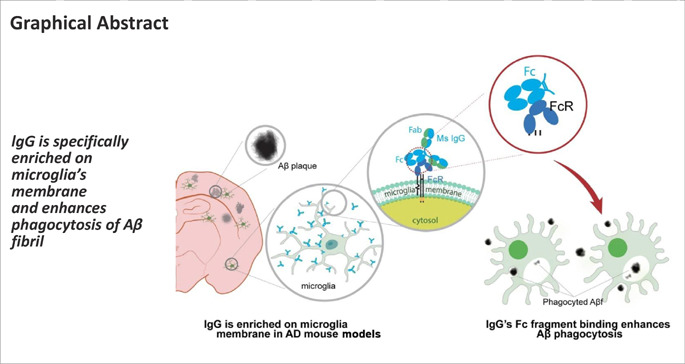

## Introduction

Alzheimer’s disease (AD) represents a significant global challenge, particularly for the aging population. In the central nervous system (CNS), AD is characterized by extracellular amyloid-beta (Aβ) deposition, intracellular tau protein accumulation resulting in neurofibrillary tangles, and brain atrophy (DeTure and Dickson, 2019). Accompanying Aβ deposition and tau accumulation are innate immune system activation and neuroinflammation, both considered pathological hallmarks of AD. As the pivotal component of the innate immune system, microglia actively patrol the brain parenchyma and respond to phagocytose Aβ plaques (Yuan et al., 2016; Grubman et al., 2021; Teng et al., 2024). However, microglia gradually shift to a disease-associated state and promote chronic neuroinflammation (Leng and Edison, 2021). In addition to microglia, adaptive immune cells may also contribute to neurodegenerative diseases (Bettcher et al., 2021; Berriat et al., 2023). The role of adaptive immunity in AD is mostly linked to T cell populations, which can directly infiltrate the brain and exert either neuroprotective or deleterious roles in AD pathology (Baruch et al., 2015; Machhi et al., 2021; Chen et al., 2023; Jorfi et al., 2023; Su et al., 2023). However, very little is known about the role of lymphatic B cells in AD progression.

Although there are several reports suggesting the potential implication of B cells in AD pathology, the percentage change of peripheral blood B cells in AD individuals and their correlation with disease severity are controversial (Richartz-Salzburger et al., 2007; Söllvander et al., 2015; Huang et al., 2022; Park et al., 2022; Wang et al., 2023). In addition, using immunodeficient mouse models of amyloid pathology, one study showed that depletion of mature B cells aggravated the Aβ load and memory deficits of mice with amyloid pathology (Marsh et al., 2016; Feng et al., 2023), whereas another study showed that B cell depletion reversed amyloid pathology (Kim et al., 2021). In particular, unlike the high numbers of T cell populations seen in the brain of AD individuals and mouse models of amyloid pathology, B lymphocytes are nearly absent in the CNS (Anthony et al., 2003; Ferretti et al., 2016; Croese et al., 2021; Jain and Yong, 2022), raising the critical question of how peripheral B cells are linked to amyloid pathology.

Gamma-type immunoglobulin (IgG), produced by B lymphocytes, has diverse antigenic specificity in the adaptive immune system, but is also present in large amounts in blood without prior known antigenic experience, and is considered to be the first line of defense against pathogens, participating in the innate immune response (Panda and Ding, 2015; Akaishi and Misu, 2024; Li et al., 2024). IgG has been successfully used to clear Aβ aggregates both in AD animal models and patients (Sevigny et al., 2016; Dubey et al., 2020; McDade et al., 2022; Tanne, 2023). This suggests a potential interaction between blood-derived IgG and the cells within the CNS parenchyma. Currently, it remains unclear how IgG influences AD pathology. Thus, determining the localization pattern and function of IgG in AD brains will be of considerable significance for understanding the interaction between B cells and innate immune cells in AD pathology.

In the present study, we explored how IgG affects AD pathology. We first determined the preferential localization of IgG to specific cell types in the brain parenchyma of multiple mouse models of AD pathology. The specific enrichment of IgG on microglia across the mouse brain with amyloid pathology led us to further determine whether this was a result of breaching the blood–brain barrier (BBB) or increased peripheral IgG level. Because microglia express Fc receptors for IgG (Doens and Fernández, 2014), which is a key player in various biological processes such as phagocytosis, we hypothesized that IgG exerts diverse influence on the microglial transcriptome, and may affect microglial phagocytosis capacity of amyloid fibrils via its interaction with microglial Fc receptors.

## Methods

### Animals

Specific-pathogen-free wild-type C57BL/6J mice (postnatal day 9 to 12 [P9–P12], body weight approximately 5 g, female, *n* = 18; P150–P180, body weight 20–25 g, male *n* = 9, female, *n* = 12) were obtained from Guangdong Medical Laboratory Animal Center (license No. SCXK (Yue) 2019-0035). 5×FAD mice (P150–P180, body weight 20–25 g; male, *n* = 15, female, *n* = 15) were originally obtained from Jackson Laboratory (Stork# 034848, RRID: MMRRC_034848-JAX, Bar Harbor, ME, USA). APP/PS1 (P150–P180; male, *n* = 3, female, *n* = 1) mice were originally obtained from Cavens (Cat# C000111, RRID: MMRRC_034829-JAX, Changzhou, China). APP^NL-G-F^ (P150–P180; male, *n* = 1, female = 2) knock-in mice were originally transferred from RIKEN Center for Brain Science (Cat# RBRC06344, RRID: IMSR_RBRC06344, Wako, Saitama, Japan). The 5×FAD, APP/PS1, and APP^NL-G-F^ mice were bred in the animal facility of Shenzhen Institute of Advanced Technology (license No. SCXK (Yue) 2021-0119). The mice were maintained at 22–25°C in 50%–60% humidity with 12-hour light and dark cycles; water and food were freely available. Animal protocols followed the Guide for the Care and Use of Laboratory Animals (National Research Council, 2011), and Shenzhen Institute of Advanced Technology Animal Care and Use Committee approved the experimental protocols (approval SIAT-IACUC-210721-YGS-XZ-A1585-01 and SIAT-IACUC-210210-YGS-XZ-A1586) on July 22, 2021.

### BBB integrity assay

Wild-type (P150–P180, *n* = 4) and 5×FAD (P150–P180, *n* = 5) mice were intravenously injected with fluorescein isothiocyanate(FITC)-dextran (MW = 150 kDa, 74817, Sigma-Aldrich, St. Louis, MO, USA) at a concentration of 20 mg/mL and a volume of 100 μL permouse via tail vein. Three hours later, the mice were anesthetized with a mixture of ketamine hydrochloride (100 mg/kg of body weight, GutianPharma, Ningde, Fujian, China) and xylazine (10 mg/kg of body weight, X1126, Sigma-Aldrich) by intraperitoneal injection, and then transcardially perfused with 0.01 M phosphate buffered saline (PBS). The brains were quickly harvested and was homogenized and the obtained supernatant was diluted 1:5 in PBS. The liquid samples were then added to a 96-well plate and examined by a plate reader (A51119600C, ThermoFisher, Waltham, MA, USA) using thee excitation/emission parameters of 490/520 nm. The obtained FITC signals were normalized by that of wild-type for statistical analysis. To study BBB integrity of 5×FAD mouse brain in more details via histological methods, another three wild-type (P150–P180) and three 5×FAD (P150–P180) mice were intravenously injected with FITC-dextran (MW = 150 kDa) at a concentration of 20 mg/mL and a volume of 100 μL via tail vein. 3 hours later, the mice were anesthetized with a mixture of ketamine hydrochloride (100 mg/kg of body weight) and xylazine (10 mg/kg of body weight) by intraperitoneal injection. The brains were quickly harvested and fixed in 4% paraformaldehyde (PFA; P6148, Sigma-Aldrich) in 0.01 M phosphate buffered saline (PBS) for 3 hours, before histological experiments.

### Enzyme-linked immunosorbent assay

For brain isolation, all animals were anesthetized with a mixture of ketamine hydrochloride (100 mg/kg of body weight) and xylazine (10 mg/kg of body weight) by intraperitoneal injection, and then perfused with 0.01 M PBS. The brain was rapidly harvested and flash-frozen with liquid nitrogen. All of the following procedures were performed on ice or at 4°C. For IgG quantification, the brain was further processed with a whole protein extraction kit (C510003-0050, Sangon, Shanghai, China) according to the manufacturer’s protocol. The obtained protein was quantified with a Pierce BCA protein assay kit (23227, ThermoFisher), and 1 μg protein per biological replicate was assayed with high-sensitivity mouse IgG enzyme-linked immunosorbent assay kit (EMC116(H).96, NeoBioscience, Shenzhen, China) according to the manufacturer’s protocol.

### Microglia isolation and culture

Postnatal mice at P9–P12 were deeply anesthetized with a mixture of ketamine hydrochloride (100 mg/kg of body weight) and xylazine (10 mg/kg of body weight) by intraperitoneal injection. After cardiac perfusion with 0.01 M PBS, brains were rapidly harvested and minced in chilled L15 medium (41300039, Thermofisher). Then, the tissue was dissociated in papain (15 U/mL, P4762, Sigma-Aldrich) for 20 minutes at 37°C and terminated by a 10% ovomucoid in L15 culture medium (2 mg/mL). Next, myelin and cell debris were removed from the single-cell suspension through the density gradient centrifugation in Percoll (25% v/v, P8370, SolarBio, Beijing, China). After thorough rinsing with ethylenediaminetetraacetic acid-free magnetic-activated cell-sorting (MACS) buffer (0.5% bovine serum albumin [BSA; A602442, Sangon] in Ca^2+^- and Mg^2+^-free PBS), the cells were incubated with microbeads conjugated with anti-CD11b antibody and purified with QuadroMACS system (130-091-051, Miltenyi, BergischGladbach, Germany) twice. The purified cells were resuspended in microglia culture medium (Dulbecco’s modified Eagle medium [DMEM]/F12 medium, C11330500CP, ThermoFisher) containing 100 U/mL penicillin plus 100 μg/mL streptomycin (15140122, ThermoFisher), 2 mM glutamine (25030081, ThermoFisher), 5 μg/mL N-acetyl cysteine (A9165, Sigma-Aldrich), 5 μg/mL insulin (16634, Sigma-Aldrich), 100 μg/mL apo-transferrin (T1147, Sigma-Aldrich), 100 ng/mL sodium selenite (S-5261, Sigma-Aldrich), 100 ng/mL mouse interleukin-34 (5195-CF, R&D Systems, Minneapolis, MN, USA), 2 ng/mL human tumor growth factor β2 (100-35B, Peprotech, Cranbury, NJ, USA), 1.5 ng/mL cholesterol (700000P, Avanti Polar Lipids, Alabaster, AL, USA), 1 μg/mL heparan sulfate (GAG-HS01, Galen Laboratory Supplies, North Haven, CT, USA), 0.1 μg/mL oleic acid (90260, Cayman Chemicals, Ann Arbor, MC, USA), and 1 ng/mL gondoic acid (354233, Cayman Chemicals, Ann Arbor, MC, USA), and inoculated onto a glass-bottom 24-well plate (coated with 2 μg/mL collagen IV [354233, Corning, New York, NY, USA]) at a density of 1 × 10^5^ cells/cm^2^. After 12 hours of incubation at 37°C with 5% CO_2_, the cells were rinsed twice with PBS and used for IgG incubation-RNA extraction or Aβ fibril phagocytosis assay. For all experiments, a minimum of three biological replicates was used with a minimum of three technical replicates per biological replicate.

Before IgG incubation with microglia, 100 mg mouse IgG (SP031, SolarBio) was first treated with an endotoxin depletion kit (ER0015, Bioendo, Xiamen, China) according to the manufacturer’s protocol. The endotoxin depleted IgG was then concentrated by Amicon Ultra centrifugal filters (MWCO 10 kDa, UFC9010, Sigma-Aldrich), and the IgG concentration was determined by a Pierce BCA assay kit (ThermoFisher), adjusted to 20 mg/mL. The endotoxin level of IgG was 0.59 EU/mL (one EU equals 0.1 ng endotoxin/mL) as determined by endotoxin assay kit (L00350, Genscript, Nanjing, China), below the 1 EU/mL standard for cell culture (Weary, 1990). The final IgG was added to fresh microglia culture medium at a concentration of 40 μg/mL, and incubated with PBS-rinsed microglia for 4 hours before cytochemistry or for 24 hours before RNA extraction. To further rule out endotoxin’s effect, the endotoxin level of the medium for all samples was equalized at 0.0012 EU/mL by adjusting with lipopolysaccharides (L2654, Sigma-Aldrich).

### Isolation of blood lymphocytes

Wild-type and 5×FAD mice (three mice each, P150–P180) were injected with 0.1 mL heparin (200 U/mL in PBS, A603251, Sangon) 1 hour before anesthetization. Blood was collected when performing cardinal puncture on the left ventricle of anesthetized mice. After centrifugation at 500 × *g* for 5 minutes and removal of the supernatant, 5 mL ACK lysis buffer (A1049201, ThermoFisher) was added to resuspend the cell pellets and lyse the red blood cells for 5 minutes on ice. A volume of 40 mL PBS was added to the cell suspension, and the cell suspension was centrifuged at 500 × *g* for 5 minutes. The obtained cell pellet was resuspended in 400 μL fluorescence-activated cell-sorting (FACS) staining buffer (ThermoFisher). B cells were isolated via B220 staining and FACS analysis as described below.

### Flow cytometry

Isolated microglia were resuspended in FACS staining buffer (ThermoFisher), and then incubated with allophycocyanin-conjugated anti-CD11b antibody (1 μg/mL, 550019, BD Biosciences, Franklin Lakes, NJ, USA) for 1 hour at 4°C. After three FACS buffer rinses, the cells were incubated with propidium iodide (PI, 50 μg/mL, P1304MP, ThermoFisher) on ice for 10 minutes to label dead cells. Flow cytometry was performed with a Wellgrow EasyCell (Wellgrow Technology, Shenzhen, Guangdong, China).

Isolated blood lymphocytes were incubated with 7-aminoactinomycin D (7-AAD; 559925, BD Biosciences) and B220-FITC (FITC-65139, ProteinTech, Wuhan, China) (both 1:100 diluted) for 1 hour at 4°C, then 1 mL FACS staining buffer was added and the cell suspension was centrifuged at 500 × *g* for 5 minutes. The cell pellets were resuspended in 3 mL FACS staining buffer and passed through a 40-μm nylon strainer (Corning). B cells were collected as the 7-AAD−B220-FITC^+^ portion on a BD FACSAria III cell sorter (**Additional Figure 1**).

### Microglia RNA sequencing and analysis

Total RNA was extracted from the cultured microglia (and isolated B cells) using TRIzol Reagent (ThermoFisher) following the manufacturer’s protocol. After that, RNA was subjected to 100-bp pair-end sequencing on the DNBSEQ platform (BGI, Shenzhen, China). At least 60 million clean reads of sequencing depth were obtained for each sample.

RNA sequencing raw data were initially filtered to obtain clean data after quality control, including removing adaptors, reads with more than 10% unknown bases, and low-quality reads. Clean data were aligned to the mouse genome (mm10) by HISAT2 (https://daehwankimlab.github.io/hisat2/). Raw counts for each gene were calculated by Htseq40 (https://htseq.readthedocs.io/en/latest/index.html; Putri et al., 2022). StringTie (https://ccb.jhu.edu/software/stringtie/) was used to estimate the expression level of detected genes. To ensure a robust analysis, genes that were detected in less than half the samples of each group or below the threshold of five reads averaged across all samples were not included in the differentially expressed gene (DEG) calculation. DEseq2 (https://bioconductor.org/packages/release/bioc/htmL/DESeq2.html) was used to evaluate the statistical significance of DEGs with raw counts. DEGs were defined as genes with a *Q*-value less than 0.05 and fold change higher than 1.5 (upregulation) or lower than 1.5 (downregulation). Gene ontology enrichment analysis was performed via the online platform (https://biosys.bgi.com) from BGI and visualized with ggplot2 (https://ggplot2.tidyverse.org/).

### Quantitative polymerase chain reaction assay

Total RNA of microglia and B cells were quantified with a NanoDrop 2000c spectrophotometer (ThermoFisher). Then RNA was reverse transcribed into complementary DNA by the Superscript IV first stand system (18091200, ThermoFisher). Quantitative polymerase chain reaction (qPCR) was then conducted by FastStart Essential DNA Green Master (06402712001, Roche, Basel, Switzerland) in the LightCycler 96 real-time PCR system (05815916001, Roche). Glyceraldehyde 3-phosphate dehydrogenase (Gapdh) was used for normalization. The primers used were listed in **Additional Table 1**. Multiple paired *t*-tests were performed to compare qPCR and RNA sequencing results. Simple linear regression was performed to test the correlation between qPCR and RNA sequencing results. All statistics were performed with RStudio 4.3 (https://posit.co/download/rstudio-desktop) and visualized with ggplot2.

**Additional Table 1:** Primers for quantitative polymerase chain reaction

Gene	Forward primer (5’→3’)	Reverse primer (5’→3’)
*C1qa*	ATGGAGACCTCTCAGGGATG	ATACCAGTCCGGATGCCAGC
*Aif1*	GACTGCCAGCCTAAGACA	AGGAATTGCTTGTTGATCCC
*Itgam*	GGCCTATACAAGCTTGGCTTT	TAGACCTGCTTGTTGCTGGG
*Trem2*	AGGGCCCATGCCAGCGTGTGG	CCAGAGATCTCCAGCATC
*C1qb*	TGGCTCTGATGGCCAACCAG	GACTTTCTGTGTAGCCCCGT
*Fcgr1*	TGCTGGATTCTACTGGTGTGA	AAACCAGACAGGAGCTGATGA
*Fcgr3*	ATCACTGTCCAAGACCCAGC	GCCTTGAACTGGCGATCCTC
*Ccr1*	TTAGCTTCCATGCCTGCCTTATA	TCCACTGCTTCAGGCTCTTGT
*Ccl7*	ATCTCTGCCACGCTTCTGTG	CCTCTTGGGGATCTTTTGTTTC
*Ptgs2*	GCTCAGCCAGGCAGCAAATC	ACCATAGAATCCAGTCCGGGT
*Socs3*	GGGTGGCA AAGAAAAGGAG	GTTGAGCGTCAAGACC CAGT
*Gapdh*	TGAGGCCGGTGCTGAGTATG	TGGTTCACACCCATCACAAACA
*Ighg1*	GTCTATCCACTGGCCCCTG	GACAGGGATCCAGAGTTCCAG
*Ighg2c*	GTCTATCCACTGGCCCCTG	GCCACATTGCAGGTGATGG
*Igh2b*	GTCTATCCACTGGCCCCTG	CACTGAGCTGCTCATAGTGTAGAGTC
*Ighg3*	GTCTATCCCTTGGTCCCTGG	CTCAGGGAAGTAGCCTTTGACA

### *In vitro* amyloid-β fibril phagocytosis assay

Aβ_42_ protein (P9001, Beyotime, Shanghai, China) was dissolved in 1 mM NaOH to a concentration of 5 mM, further diluted to 100 μM with 10 mM hydrochloric acid, and then incubated at 37°C for 24 hours. The obtained Aβ fibrils were labeled with Pierce FITC antibody labeling kit (53027, ThermoFisher) or pHrodo^TM^ iFL Red STP ester (P36011, ThermoFisher) according to the manufacturer’s protocol. The obtained FITC- or pHrodo-Aβ fibril solution was diluted in DMEM/F12 to 1 μM as working concentration and added to PBS-rinsed microglia.

To investigate the role of the IgG Fc fragment in the enhancement of microglial phagocytic capacity, goat anti-mouse IgG (M4280, Fc specific, Sigma-Aldrich) and goat anti-mouse IgG (M4155, Fab specific, Sigma-Aldrich) were dialyzed with Amicon Ultra centrifugal filters (MWCO 10 kDa, Sigma-Aldrich) to remove perseverant, and incubated with mouse IgG (40 μg/mL) at a concentration of 200 μg/mL in DMEM/F12 at 37°C for 1 hour before being added to PBS-rinsed microglia.

To perform the Aβ fibril phagocytosis assay on cultured microglia, 1 μM Aβ fibril with or without mouse IgG or IgG-protein complex (anti-Fc-IgG, and anti-Fab-IgG) were added to PBS-rinsed microglia, incubated at 37°C and 5% CO_2_ for 4 hours in DMEM/F12, and fixed with 4% room temperature PFA solution for 15 minutes before immunocytochemistry.

To perform the Aβ fibril phagocytosis assay via flow cytometry, primary microglia after MACS sorting were directly incubated with 1 μM FITC-Aβ fibril with or without IgG diluted in DMEM/F12 in a non-treated 24-well plate, and incubated at 37°C and 5% CO_2_ for 4 hours in DMEM/F12. Cells were then collected and washed twice via centrifugation. The cell suspension was then stained with PI (50 μg/mL) and underwent FACS using a Wellgrow EasyCell. The ratio of FITC^+^ to PI^−^ cells was calculated for the IgG-treated microglia group and non-treated group.

### Immunohistochemistry and immunocytochemistry

For immunohistochemistry, all adult mice were deeply anesthetized with a mixture of ketamine hydrochloride (100 mg/kg of body weight) and xylazine (10 mg/kg of body weight) by intraperitoneal injection. Animals were then sequentially perfused with 0.01 M PBS and 4% PFA in 0.01 M PBS. Brains were carefully harvested, followed by postfixation in 4% PFA in 0.01 M PBS. Brains were cryoprotected in 30% sucrose in 0.01 M PBS at 4°C for 2–3 days. After being embedded in optimal cutting temperature compound (Tissue-Tek, Sakura Finetek, Torrance, CA, USA), brain samples were frozen in liquid nitrogen and stored at –80°C before sectioning. Tissue with regions of interest were cut with a cryostat (Leica, Wetzlar, Germany) at a thickness of 30 μm. Brain sections were rinsed with PBS three times for 10 minutes each, followed by incubation in 2% protein-free blocking solution containing 0.5% Triton X-100 (T8787, Sigma-Aldrich) at room temperature (25°C) for 1 hour. Then, samples were incubated with primary antibodies with 1% protein-free blocking solution at 4°C overnight. After three PBS rinses, samples were incubated with Alexa Fluor-conjugated secondary antibodies (ThermoFisher) in 1% protein-free blocking solution with 4′,6-diamidino-2-phenylindole (DAPI; 1:1000, MBD0015, Sigma-Aldrich) at room temperature for 2 hours. Then, samples were rinsed with three washes before mounting with ProLong Gold antifade mountant (P36930, ThermoFisher).

For immunocytochemistry, the PFA-fixed microglia were rinsed twice with PBS for 5 minutes each, followed by 2% protein-free blocking solution (C530040, Sangon) containing 0.5% Triton X-100 at room temperature for 1 hour. Then, cells were incubated with primary antibodies with 2% protein-free blocking solution containing 0.5% Triton X-100 at 4°C overnight. After three PBS rinses, samples were incubated with Alexa Fluor-conjugated secondary antibody (ThermoFisher) in 1% protein-free blocking solution with DAPI (1:1000) at room temperature for 2 hours. Then, samples were rinsed twice before mounting with custom-made antifade mountant (one-part 0.1 M PBS solution, nine-parts glycerol [1.04095, Sigma-Aldrich], 0.1-part 20% n-propyl gallate [02370, Sigma-Aldrich]). Antibodies used were listed in **Additional Table 2**.

**Additional Table 2:** Antibodies for immunohistochemical and immunocytochemical stainings

Antibody	Working dilution	Supplier	Catalog number	RRID
***Primary antibodies***				
Anti-Mouse IgG (Fc specific) antibody	1:2000	Sigma-Aldrich (St. Louis, MO, USA)	M4280	AB_260521
Anti-Mouse IgG (Fab specific) antibody	1:2000	Sigma-Aldrich	M4155	AB_260514
Recombinant anti-TMEM119 antibody [28-3] - Microglial marker	1:500	Abcam (Cambridge, UK)	ab209064	AB_2800343
AIF1/IBA1 antibody, Rabbit Polyclonal	1:1000	AffBiotech (Cincinnati, OH, USA)	DF6442	AB_2838405
Anti Iba1, Rabbit	1:1000	Wako (Richmond, VA, USA)	019-19741	AB_839504
***Secondary antibodies***				
Donkey anti-Mouse IgG (H+L) Highly Cross-Adsorbed Secondary Antibody, Alexa Fluor™ 488	1:500	ThermoFisher (Waltham, MA, USA)	A-21202	AB_141607
Donkey anti-Rabbit IgG (H+L) Highly Cross-Adsorbed Secondary Antibody, Alexa Fluor™ 568	1:500	ThermoFisher	A10042	AB_2534017
Donkey F(ab')2 Anti-Mouse IgG H&L (Alexa Fluor® 488)	1:500	Abcam	ab181289	AB_2813900
Donkey anti-Goat IgG (H+L) Cross-Adsorbed Secondary Antibody, Alexa Fluor™ 568	1:500	ThermoFisher	A-11057	AB_2534104
Donkey anti-Rat IgG (H+L) Highly Cross-Adsorbed Secondary Antibody, Alexa Fluor™ 568	1:500	ThermoFisher	A78946	AB_2910653

### Microscopy and image analysis

Confocal images of fluorescent specimens from brain and microglia were taken with an Olympus IXplore SpinSR confocal microscope (Olympus, Tokyo, Japan) equipped with solid state lasers. Extended Apochromat 10× (0.4 NA, air), 40× (1.4 NA, air), and 60× (1.42 NA, oil) objectives were used. Z-stacked focal plans were taken and maximal-projected with cellSens (Olympus); brightness and contrast were adjusted if necessary. Four regions were randomly chosen and photographed with a scientific CMOS camera (Hamamatsu, Hamamatsu City, Japan) per mouse sample from different groups based on the target protein’s labeling fluorescence wavelength. Super-resolution images of microglia were taken with a Carl Zeiss LSM 900 confocal microscope (Carl Zeiss, Jena, Saxe-Weimar-Eisenach, Germany) equipped with AiryScan 2 module, via a Plan-Apochromat 63× objective (NA 1.4), with filter level set to high. Microglial terminals were photographed using the same setting, except that a zoom factor of 5 instead of 1.3 was used.

For fluorescence quantification, the images were converted from 16-bit to 8-bit format, and the average grayscale values across the individual images were calculated in FIJI 2 (Schindelin et al., 2012) for comparison. The fluorescent profile was plotted with FIJI software. For a selected region of interest, the plot displayed a “column average plot,” where the *x*-axis represented the horizontal distance through the selection and the *y*-axis represented the vertically averaged pixel intensity.

For 3D reconstruction of various cell types and IgG signal colocalization, the fluorescent images were binarized by an auto-threshold algorithm, and 3D models were constructed with Volume Viewer in FIJI. For 3D visualization of colocalization between brain cell type-specific markers and IgG, binarized image stacks of brain cell markers and IgG underwent AND logical operation, and the obtained image stacks were constructed into 3D models via Volume Viewer. The voxels with colocalization signals were counted, divided by total voxel number, and compared across various brain cell types.

### Statistical analysis

For all experiments, at least three biological replicates were used with a minimum of two technical replicates for each biological replicate. Four randomly chosen views of field were analyzed for each biological replicate to account for variability within the sample itself. The ratio of target cells to total ionized calcium binding adaptor molecule 1-positive (Iba1^+^) microglia cells was calculated. The Welch and Brown–Forsythe versions of one-way analysis of variance with Dunnett’s multiple comparison test were performed when comparing multiple groups against a single treatment. A two-tailed Welch’s *t*-test assuming unequal variances was used when only two groups were analyzed. For all experiments, statistical significance was determined by *P*-values < 0.05 in Rstudio.

## Results

### IgG is abundantly present in 6-month-old 5×FAD mouse brains without IgG leakage from blood–brain barrier or B cell infiltration

**Figure 1:**
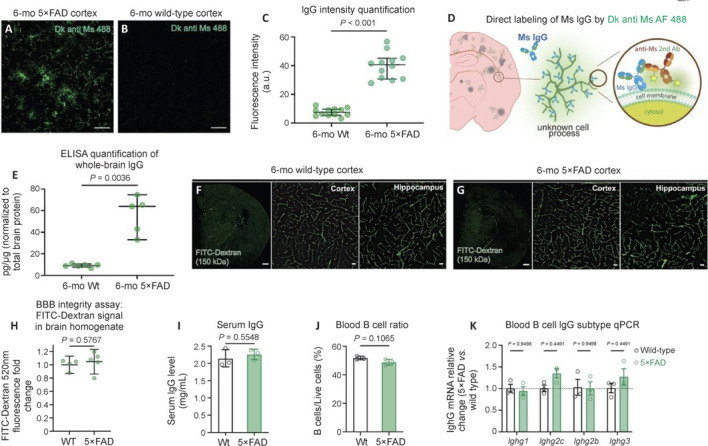
IgG is accumulated in 5×FAD mouse brains and localizes to unknown cell processes. (A, B) With no prior primary antibody incubation, direct incubation with an Alex-flour 488-labeled antibody against mouse IgG (Dk anti-Ms AF488) in brain sections for 12 hours showed apparent IgG signal on unknown cell processes in the 5×FAD mouse brain (A), but not wild-type mouse brain (B). (C) The IgG fluorescence intensity quantification in microglia showed a difference between wild-type and 5×FAD mice (*P* < 0.0001). (D) Diagram illustrating our hypothesis that mouse IgG would be present in unknown cell processes recognized by secondary antibodies against mouse IgG. (E) ELISA quantification of IgG level in brain lysate of wild-type and 5×FAD mice (*P* = 0.0036). (F–H) The 6-month-old 5×FAD mouse brain maintained its integrity, as indicated by immunohistochemistry (F, G) and spectrophotometry (H) after a single dose of 150 kDa FITC-dextran i.v. injection. Scale bars: 30 μm. (I) ELISA quantification of serum IgG in 6-month (mo)-old wild-type and 5×FAD mice. (J) The blood B cell ratio of 6-month-old wild-type and 5×FAD mice. The FACS result was shown in Additional Figure 1. (K) The relative qPCR quantification of different IgG subtype mRNAs in serum B cells of wild-type and 5×FAD mice. Data are expressed as mean with 95% confidence interval (CI), and were analyzed by two-tailed Welch’s *t*-test. 5×FAD: Familial Alzheimer’s disease mutations; BBB: blood–brain barrier; Dk: donkey; ELISA: enzyme-linked immunosorbent assay; FACS: fluorescence-activated cell sorting; FITC: fluorescein isothiocyanate; IgG: gamma-type immunoglobulin; i.v.: intravenous; mo: month; Ms: mouse.

When we directly incubated anti-mouse secondary antibody to probe 6-month-old 5×FAD mouse brains, we found there were immunoreactive signals in unknown cell processes of 6-month-old 5×FAD mouse brains that were not found in wild-type mouse brains (**Figure 1A–C**). This unusual pattern led us to hypothesize that the unknown cell processes were enriched with mouse IgG per se, so that anti-mouse secondary antibodies recognized these anchored mouse IgG and labeled the unknown cell processes (**Figure 1D**). To test this, we performed enzyme-linked immunosorbent assays to quantify IgG of the whole brain. IgG was abundantly present in 6-month-old 5×FAD mouse brains (**Figure 1E**).

We next investigated whether the presence of parenchymal IgG in 6-month-old 5×FAD mouse brains was due to disruption of the BBB. Compared with normal mouse brains, 6-month-old 5×FAD mouse brains showed no apparent BBB permeability to 150 kDa FITC-dextran, which is comparable to IgG in molecular size (**Figure 1F** and **G**). In agreement with previous studies (Bien-Ly et al., 2015; Marsh et al., 2016), our quantitative analysis of FITC-dextran that was leaked into brain parenchyma showed no difference between normal and 5×FAD mouse brains (**Figure 1H**), indicating that the observed parenchymal IgG did not leak from a damaged BBB. Because there are few or no peripheral B cells that infiltrate normal or AD brains in humans and mice (Anthony et al., 2003; Ferretti et al., 2016; Croese et al., 2021; Jain and Yong, 2022), it is unlikely that the abundant IgG in brain parenchyma was produced by locally infiltrated B cells, if there are any. We next examined whether IgG presence in the brain was due to IgG elevation in the serum of the 5×FAD mice. The serum IgG level of 5×FAD was not significantly different compared with that of the wild-type mice (**Figure 1I**), nor was the blood B cell number (**Figure 1J** and **Additional Figure 1**). In addition, the mRNA levels of four IgG subtypes expressed by blood B cells showed no significant differences between wild-type and 5×FAD mice (**Figure 1K**). The findings suggested that IgG appeared to be enriched in some unknown cell processes, but it remains unclear how IgG accumulates in 5×FAD mouse brains where B cells are virtually absent and the BBB remains nonpermeable to IgG.

### Parenchymal IgG is specifically enriched on microglia in 5×FAD mouse brains

**Figure 2:**
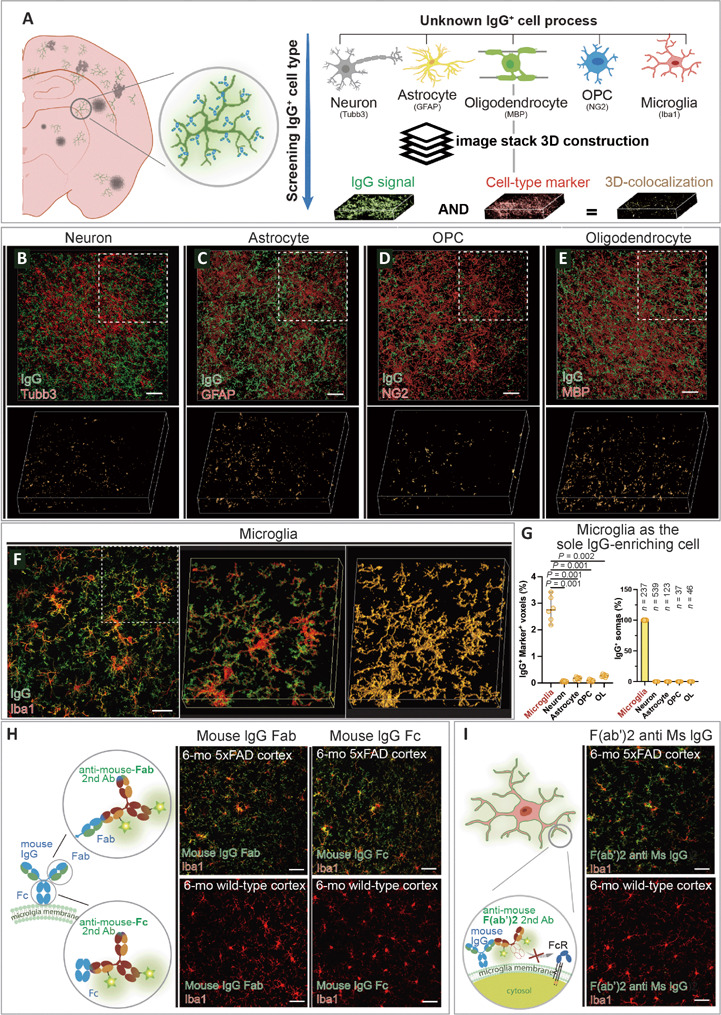
Microglia are the only cell type enriched with IgG in 6-month-old 5×FAD mouse brain. (A) Diagram depicting the strategy to identify the brain cell type enriched with IgG via 3D reconstruction. Brain cell types were stained with corresponding markers and imaged with confocal z-stacks for 3D reconstruction in FIJI. (B–E) IgG was not enriched on neurons, astrocytes, oligodendrocyte precursor cells, or mature oligodendrocytes, which were labeled by Tubb3 (red, Alexa Fluor 488), GFAP (red, Alexa Fluor 488), NG2 (red, Alexa Fluor 488) and MBP (red, Alexa Fluor 488), respectively. The magnified images in the dashed boxes show IgG colocalization with each marker. (F) IgG was enriched on microglia. The magnified images in the dashed box show IgG colocalization with the microglial marker. (G) Microglia was the only brain cell type that was enriched with IgG, as showed by quantification of the IgG^+^marker^+^ voxel ratio and IgG^+^marker^+^ cell soma ratio. Data are expressed as mean with 95% CI and were analyzed by one-way analysis of variance followed by Dunnett’s multiple comparison test. (H) The antibodies against mouse IgG Fab and Fc fragments showed that IgG was enriched on microglia in 5×FAD mice. (I) Fc-deficient F(ab’)2 antibody against mouse IgG showed that IgG was enriched on microglia in 5×FAD mice. Scale bars: 500 μm (low magnification images of brain sections in B, D, F and H), 30 μm (others). 5×FAD: Familial Alzheimer’s disease mutations; Fab: fragment antigen-binding; Fc: fragment crystallizable; Iba1: ionized calcium binding adaptor molecule 1; IgG: gamma-type immunoglobulin; GFAP: glial fibrillary acidic protein; MBP: myelin basic protein; mo: month; NG2: neural/glial antigen 2; OPC: oligodendrocyte progenitor cell; Tubb3: class III β-tubulin.

Next, we focused on determining the origin of the IgG-enriched cell processes. We performed a whole-brain cell-type screening to identify the origin of cell processes, including astrocytes, microglia, neurons, oligodendrocytes and oligodendrocyte precursor cells, that are known to contain cell processes or branches (**Figure 2A**). Astrocytes, neurons, oligodendrocytes, and oligodendrocyte precursor cells showed very little or no IgG enrichment (**Figure 2B–E** and **G**). Microglia showed high levels of colocalization with IgG signals (**Figure 2F**), and every examined Iba1^+^ microglial soma displayed IgG signal (**Figure 2G**), indicating the cell processes displaying IgG signal solely originated from microglia.

To further determine that IgG was indeed associated with microglia in the 5×FAD mouse strain, several primary antibodies against mouse IgG were tested. The IgG can be cleaved into two fragments by papain treatment: Fab and Fc. Antibodies against both mouse IgG Fab and Fc fragments confirmed that microglia were enriched with IgG in 5×FAD mice (**Figure 2H**). Because microglia express Fc fragment receptors (FcR), it was necessary to rule out the possibility that microglia nonspecifically bound the Fc fragments of any secondary antibodies. We first confirmed that whole-molecule secondary antibodies (Fab + Fc) against IgG from other species, such as rats, did not produce any signals associated with microglia (**Additional Figure 2**). Furthermore, we tested Alexa Fluor-conjugated F(ab’)2 against mouse IgG, a type of secondary antibody lacking the Fc fragment, which also stained microglial processes (**Figure 2I**). These results confirmed that 5×FAD mouse microglia actually bind mouse IgG, and that the findings were not due to nonspecific interaction between microglial FcRs and Fc fragments of secondary antibodies. Taken together, we demonstrated that the abundant IgG in brain parenchyma of 5×FAD mouse strain of amyloid pathology showed enrichment specificity on microglia.

### Microglia are enriched with IgG across the whole brain in different mouse models of amyloid pathology

**Figure 3:**
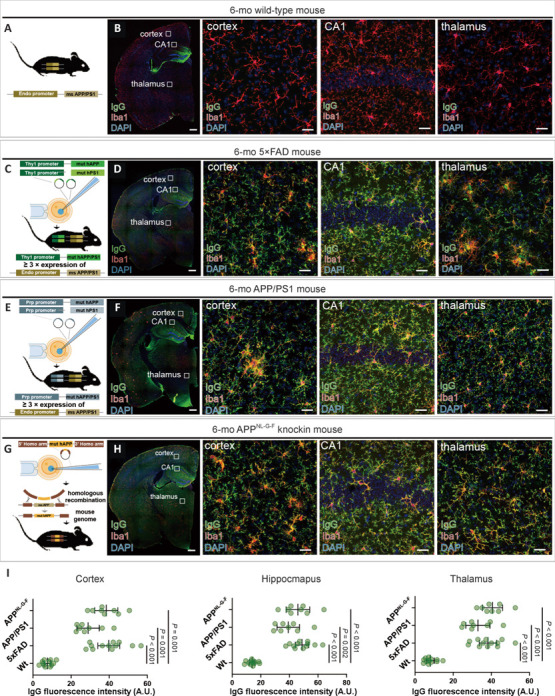
IgG is enriched on microglia across the whole brain in multiple mouse models of amyloid pathology. (A) Diagram depicting wild-type mouse APP and PS1 endogenous gene expression regulated by their respective native genomic promoters. (B) The wild-type mouse microglia (Iba1, Alexa Fluor 568) had weak IgG signals across the whole brain. The regions of cortex, CA1 hippocampus, and thalamus were enlarged. (C) Diagram showing the genetics of the 5×FAD mouse strain. The mutated human APP and PS1 transgenes were overexpressed under the control of mouse Thy1 promoter. (D) The microglia were enriched with IgG across the whole brain in the 5×FAD mouse. The regions of cortex, CA1 hippocampus, and thalamus were enlarged. (E) Diagram depicting the genetics of the APP/PS1 mouse strain. The mutated human APP and PS1 transgenes were overexpressed under the control of mouse prion promoter. (F) The microglia were enriched with IgG across the whole brain in the APP/PS1 mouse. The regions of cortex, CA1 hippocampus, and thalamus were enlarged. (G) Diagram depicting the genetics of the APP^NL-G-F^ knock-in mouse strain. The mutated human APP transgene was introduced by homologous recombination to replace the mouse APP gene. This humanized strain avoids APP overexpression. (H) The microglia were enriched with IgG across the whole brain in the APP^NL-G-F^ knock-in mouse. The regions of cortex, CA1 hippocampus, and thalamus were enlarged. Scale bars: 500 μm (low magnification images of brain sections in B, D, F, and H), 30 μm (others). (I) The statistical results showed that IgG was enriched on microglia across the whole brain in different mouse models of amyloid pathology. Data are expressed as mean with 95% CI and were analyzed by one-way analysis of variance followed by Dunnett’s multiple comparison test. 5×FAD: Familial Alzheimer’s disease mutations; APP: amyloid precursor protein; DAPI: 4′, 6-diamidino-2-phenylindole; Iba1: ionized calcium binding adaptor molecule 1; IgG: gamma-type immunoglobulin; mo: month; PS1: presenilin 1.

Although the wild-type mouse microglia showed very little IgG signal (**Figure 3A**, **B** and **I**), IgG enrichment on microglia was seen across the entire 5×FAD brain, including cortex, hippocampus and thalamus (**Figure 3C**, **D** and **I**), indicative of a brain-wide event in amyloid pathology. To investigate whether the IgG-enriched microglia were specific to the 5×FAD mouse strain or could be seen in other models of amyloid pathology, we used two other well-characterized mouse models of amyloid pathology: APP/PS1 mouse strain and APP^NL-G-F^ knock-in mouse strain. Constructed with an overexpression strategy (**Figure 3C**; Oakley et al., 2006) similar to the 5×FAD mouse strain, the APP/PS1 transgenic strain overexpresses human pathological APP and PSa proteins that drive cognitive and behavioral impairment in mice (**Figure 3D**; Jankowsky et al., 2004). We found IgG enrichment on microglia was replicated in the APP/PS1 mouse strain (**Figure 3E**, **F** and **I**). To avoid any nonphysiological consequences that might be caused by the overexpression of APP and PS1 or non-Aβ fragments in the mouse brain, we used an APP^NL-G-F^ knock-in model that “humanized” the murine Aβ sequence (**Figure 3G**) (Saito et al., 2014). We found that IgG-enriched microglia were also present in the APP^NL-G-F^ knock-in model (**Figure 3H** and **I**), supporting that overexpression of human APP proteins did not cause IgG enrichment on microglia. Hence, these consistent observations from different mouse models of amyloid pathology support that IgG enrichment on microglia across the whole brain is a universal event that may be implicated in amyloid pathology.

### IgG binds to microglia *in situ* and *in vitro*

**Figure 4:**
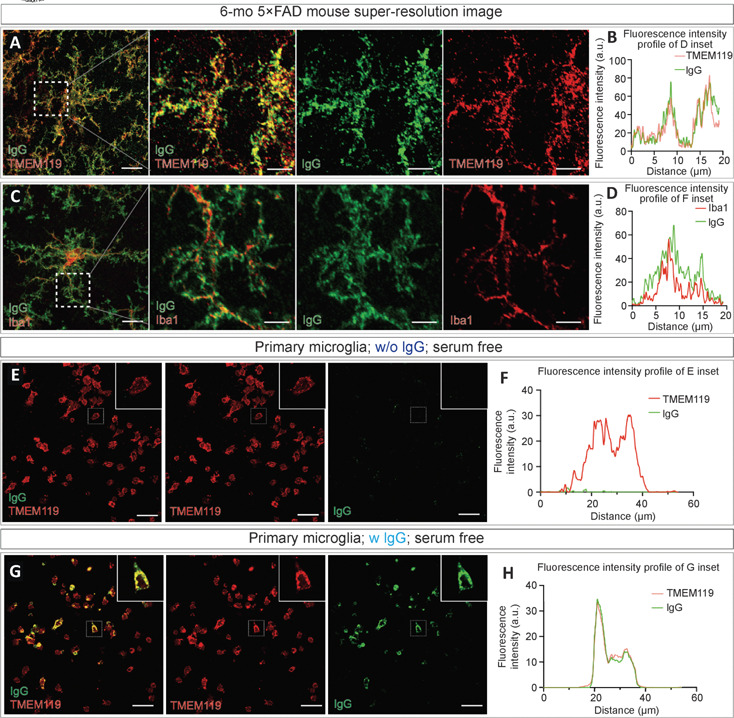
IgG binds the microglial membrane both *in situ* and *in vitro*. (A) Super-resolution images of microglia labeled with antibodies against mouse IgG (green, Alexa Fluor 488) and TMEM119 (red, Alexa Fluor 568). The enlarged cell process terminals were independently imaged. (B) Immunofluorescence intensity profile of the cell process terminals in A. (C) Super-resolution images of microglia labeled with antibodies against mouse IgG (green, Alexa Fluor 488) and Iba1 (red, Alexa Fluor 568). The enlarged cell process terminals were independently imaged. (D) Fluorescence intensity profile of the cell process terminals in C. (E) The representative images showing no IgG association with the membrane (TMEM119, red, Alexa Fluor 568) of purified microglia after *in vitro* incubation without IgG (w/o IgG) for 24 hours. (F) Fluorescence intensity profile of the inset cell from E showing no colocalization between TMEM119 and IgG due to low IgG signal. (G) Representative images showing strong IgG association with the membrane (TMEM119, red, Alexa Fluor 568) of most purified microglia after *in vitro* incubation with IgG (w IgG) for 3 hours and subsequent thorough rinsing. (H) Fluorescence intensity profile of the inset cell from G showing strong colocalization between TMEM119 and IgG. Scale bars: 10 μm (whole microglia in A and C), 3 μm (microglia terminals in A and C), 30 μm (E and G). Iba1: Ionized calcium binding adaptor molecule 1; IgG: gamma-type immunoglobulin; mo: month; TMEM119: transmembrane protein 119.

To further investigate the IgG enrichment on microglia in mouse brains of amyloid pathology, we next performed detailed examination of IgG localization on microglia via super-resolution microscopy. IgG was enriched on the cell membrane but not cytoskeleton, as indicated by colocalization with microglia-specific membrane protein transmembrane protein 119 (TMEM119) (**Figure 4Α** and **Β**) and cytoskeleton-related marker Iba1 (**Figure 4C** and **D**), respectively. On the basis of these subcellular results showing that IgG was enriched on the microglial cell membrane, we hypothesized that IgG binds to microglia directly.

To test this, we used an *in vitro* serum-free culture system that was devoid of IgG. Highly purified microglia were isolated and verified (**Additional Figure 3A–C**), then cultured in serum-free medium that lacked IgG. Microglia cultured in serum-free medium for 24 hours appeared healthy and showed very little association with IgG (**Figure 4E** and **F**). In contrast, exogenous supplementation of 40 μg/mL mouse IgG into the serum-free microglial culture readily facilitated IgG association with microglia (**Figure 4G** and **H**) and resembled the IgG enrichment in microglia from mouse models of amyloid pathology. The *in vitro* binding between IgG and microglia may be an underlying mechanism for the IgG enrichment in microglia. Notably, these binding assays, which were performed in the absence of AD-like pathology, suggest that IgG’s binding to microglia is an intrinsic property of microglia, irrespective of AD pathological conditions.

### Diverse effects of IgG on microglia gene expression

**Figure 5:**
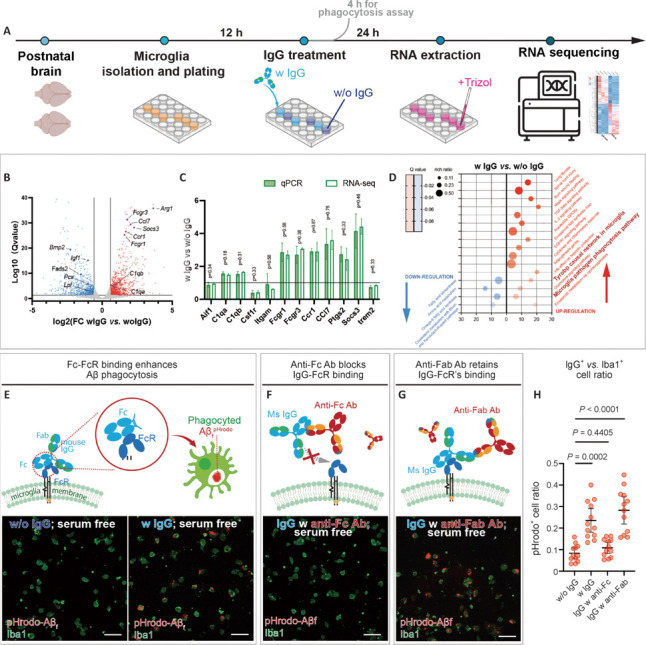
IgG enhances primary microglial phagocytosis of Aβ fibril. (A) RNA sequencing workflow to examine IgG’s effect on primary microglia. (B) Volcano map depicting DEGs in IgG-treated microglia compared with untreated naïve microglia. The designated genes are related to microglial phagocytosis, wound healing, amino acid and lipid biosynthesis and metabolism. (C) Fold changes of representative genes by qPCR and RNA sequencing of primary microglia with or without IgG treatment. Multiple paired *t*-tests were performed. *n* = 3 for IgG-treated and non-treated microglia. (D) The gene ontology analysis showed that besides the injury/wound healing pathway and cytokine/chemokine-related pathway, the microglial phagocytosis pathway was enriched in the IgG-treated group (highlighted by red, bold fonts). (E) Diagram and representative images showing IgG binding to membrane FcR enhanced microglial phagocytic capacity for Aβ fibrils. (F) Diagram and representative images showing blocking mouse IgG Fc fragment with Fc-specific antibody inhibited IgG binding to microglial FcR and abolished IgG-induced enhancement of microglial phagocytosis of Aβ fibrils. (G) Diagram and representative image showing blocking mouse IgG Fab fragment with Fab-specific antibody did not interfere with IgG binding to FcR and preserved the IgG-induced enhancement effect. (H) Quantification of the above effects by calculating the pHrodo^+^/Iba1^+^ cell ratio in different groups. Data are expressed as mean with 95% CI and were analyzed by one-way analysis of variance followed by Dunnett’s multiple comparison test. Aβf: Amyloid-beta fibril; DEGs: differentially expressed genes; FcR: Fc receptor; Iba1: ionized calcium binding adaptor molecule 1; IgG: gamma-type immunoglobulin; qPCR: quantitive polymerase chain reaction.

The spatial adjacency of IgG and microglia suggested that IgG may affect microglial function in AD conditions. To assess the impact of IgG on microglia, we isolated highly purified microglia from postnatal mice, treated them with IgG, and analyzed their transcriptomic data (**Figure 5A**). Of 12,591 identified genes (none belonging to the immunoglobulin gene family), 1831 genes displayed differential expression in microglia, distinctly segregating samples into two groups based on IgG treatment (**Additional Figure 3D–F**). The 944 genes upregulated by IgG treatment included the genes encoding complement proteins such as C1qa and C1qb, and Fc receptors (**Figure 5B**).

We validated the upregulated genes from our RNA sequencing results by conducting qPCR assays on various microglial markers (including *Aif1*, *Csf1r*, *Itgam*, *Fcgr1*, and *Trem2*) and genes encoding complements and Fc receptors, including *C1qa*, *C1qb*, *Fcgr1*, and *Fcgr3* (**Figure 5C** and **Additional Figure 3G**). Because these proteins are associated with antibody-dependent cellular phagocytosis (ADCP), we postulated that IgG treatment might enhance the phagocytic capacity of microglia.

Further, gene ontology analysis showed enrichment not only in pathways associated with injury/wound healing, cytokines/chemokines, and lipid metabolism, but also in microglia-related phagocytosis pathways within the IgG-treated group (**Figure 5D**). These results suggest that IgG exerts diverse effects on microglial gene expression, particularly in pathways relevant to phagocytosis that are significantly implicated in AD pathology.

### IgG enhances microglial phagocytic capacity for Aβ fibrils via Fc fragment

To investigate the role of IgG in AD pathology, we assessed its impact on the phagocytic capacity of microglia for Aβ fibrils *in vitro*. Primary microglia were exposed to FITC-labeled Aβ fibrils (1 μM), and the effect of IgG treatment on the ratio of FITC^+^ microglia to Iba1^+^ microglia was evaluated. The presence of 40 μg/mL mouse IgG increased the ratio of FITC^+^ microglia, as confirmed by both cytochemistry and flow cytometry (**Additional Figure 4A–D**).

Given that effective phagocytosis requires an acidic lysosome environment, we labeled Aβ fibrils with pHrodo Red, a fluorescent dye that does not fluorescence at neutral pH but becomes increasingly fluorescent as pH decreases, to track the functional phagocytosis and degradation of labeled Aβ fibrils. Treatment with mouse IgG (40 μg/mL) enhanced the delivery of Aβ fibrils into microglial acidified lysosome compartments (**Figure 5E**). These results strongly indicated the enhancement of microglial phagocytic capacity for Aβ fibrils by IgG *in vitro*.

As microglia express IgG Fc fragment receptors (FcRs) (Fuller et al., 2014), mediators of classical immune responses such as ADCP, we suspected that the Fc fragment was responsible for this effect. To test this, we blocked the mouse IgG Fc fragment using a goat-derived polyclonal antibody. Under this condition, the mouse-goat-IgG complex failed to enhance the microglial phagocytic capacity for Aβ fibrils (**Figure 5F** and **H**). However, incubating mouse IgG with a goat-derived polyclonal antibody against the mouse Fab fragment failed to abrogate microglial phagocytosis of Aβ fibrils (**Figure 5G** and **H**), indicating that the Fab fragment did not contribute to IgG’s effect on microglia. These findings support a critical role of the Fc, but not Fab, fragment of IgG in enhancing microglial phagocytosis of Aβ fibrils.

## Discussion

The interplay between the CNS and peripheral immune system, particularly in major neurological disorders such as AD, has become a central focus for researchers across disciplines (Bettcher et al., 2021; Leng and Edison, 2021). Our study sheds light on the interaction between IgG, a component of the adaptive immune system, and microglia, the primary immune cells within the CNS. We observed that in mouse models of amyloid pathology, microglia were enriched with membrane-bound IgG, and IgG significantly boosted microglial capacity for phagocytosis of Aβ fibrils *in vitro*. This enhancement relied on the interaction between IgG’s Fc fragment and the FcR, a finding substantiated by transcriptomic analysis. These outcomes suggest a potentially beneficial role of parenchymal IgG in mitigating AD pathology.

Previous studies have suggested the presence of IgG on plaque-associated Iba1^high^ microglia (Marsh et al., 2016; Kim et al., 2021), but our study has several novel findings. First, we showed that IgG was present in 6-month-old mouse models of amyloid pathology throughout all brain regions. Intriguingly, IgG is generally thought not to penetrate normal brain or even 6-month-old 5×FAD mouse brain, in which the BBB remains generally intact (Bien-Ly et al., 2015; Brigas et al., 2021). B cells are nearly absent from normal and AD brains (Anthony et al., 2003; Ferretti et al., 2016; Croese et al., 2021; Jain and Yong, 2022), refuting the possibility that the abundant IgG is locally produced by infiltrated B cells. Thus, the origin of parenchymal IgG remains unclear. Second, we showed that IgG was enriched on all microglia in multiple mouse strains of amyloid pathology, including the APP^NL-G-F^ knock-in mouse strain. Third, we showed for the first time that IgG was enriched on the microglial cell membrane, as demonstrated by its colocalization with microglial membrane protein TMEM119, but not the cytoskeleton-related classic microglia marker Iba1. Fourth, we demonstrated that microglia can bind IgG even without AD-related pathological stimulation, which may suggest that microglia have the intrinsic ability to bind IgG, possibly through its FcRs. Lastly, we provided evidence that the Fc but not Fab fragment was required for IgG’s effects on microglial phagocytic capacity for Aβ fibrils. These findings prompt renewed interest in understanding the role of IgG in AD pathology.

To decipher the mechanism underlying IgG’s enhancement of microglial phagocytic capacity of Aβ fibrils, we focused on exploring the possibility of ADCP (Cao et al., 2022). The data obtained via blocking the Fc and Fab fragments with specific antibodies echoed the mechanisms of ADCP. However, the specific mechanisms by which IgG interacts with microglia, whether via opsonization of Aβ fibrils or internalization and initiating an unknown signaling pathway, remain unclear. Advanced imaging methods, such as transmission electron microscopy or MINFLUX microscopy, are essential to discern IgG’s aggregation state, its association with Aβ fibrils, and its interaction with microglial FcR.

Our *in vitro* results showed that IgG enhanced microglial phagocytic capacity for Aβ fibrils via the Fc fragment and FcR. In situ evidence and the use of FcR knock-out animal models are essential to substantiate these findings. Furthermore, it is plausible that IgG may also bind to other targets, such as damaged neurons in later stages of AD pathology, potentially initiating antibody-dependent cellular cytotoxicity. However, verifying this would necessitate an AD animal model with obvious neuron loss.

This study focused on the impact of IgG on microglial phagocytic capacity of Aβ fibrils *in vitro*. Future investigations should prioritize elucidating the origin of brain parenchymal IgG, its transportation routes, and the factors initiating its entry into the CNS in AD pathology. There are several possibilities for the origin of IgG present in brain parenchyma in mice models of amyloid pathology. Firstly, brain barrier structures (BBB, cerebrospinal fluid–brain barrier and meninge barrier) may be breached in 5×FAD mice, which would allow IgG to diffuse freely from serum to brain parenchymal. Secondly, B cells, the primary lymphocyte type generating soluble IgG, have the potential to infiltrate brain parenchyma under pathological conditions (Comi et al., 2021). Thirdly, the IgG transportation system on brain barriers may become activated by pathological stimulation during amyloidosis. Not all of the mentioned possibilities withstand scrutiny. Firstly, the BBB remains functional in mouse models of amyloid pathology (Bien-Ly et al., 2015; Brigas et al., 2021), which excludes the possibility of IgG passive diffusion. Secondly, although B cells have been observed in both normal and mouse brains of amyloid pathology (Kim et al., 2021; Feng et al., 2023), the localization of these B cells within the brain parenchyma remains contentious. The functional barriers in the AD brain suggest that blood-derived B cells might be sequestered by these barriers, inhibiting them from secreting IgG in situ. This perspective is supported by the lack of clinical reports documenting the presence of B cells in normal or AD patient brains (Anthony et al., 2003; Ferretti et al., 2016; Croese et al., 2021; Jain and Yong, 2022). However, contrasting studies have reported the presence of B cells in single-cell RNA sequencing data (Keren-Shaul et al., 2017) and flow cytometry data of AD animal model brains (Feng et al., 2023), though in extremely low abundance. Much of the controversy arises from technical problems such as blood contamination. To clarify the origin of B cells in the AD brain, we need careful discrimination between contaminating blood cells and valid brain parenchymal B cells, and objective quantification methods such as flow cytometry and 3D imaging of whole mouse brains (Ueda et al., 2020). Establishing the presence B cells in brain parenchyma is essential for delineating the origin of parenchymal IgG and potential novel pathological mechanisms in the context of AD. Thirdly, although IgG faces restricted diffusion across the BBB in mouse models of amyloid pathology, it can potentially use the BBB’s transporting system (Alajangi et al., 2022). This system’s affinity for IgG might be augmented by AD pathology. However, a comprehensive understanding of the precise transportation route of IgG necessitates further investigation. Additionally, other brain barrier structures, such as the choroid plexus, exhibit noticeable IgG signals, vying for the pivotal regulators of IgG transportation in the mouse brain of amyloid pathology (Marsh et al., 2016). Exploring these potential avenues is intricately linked to identifying the AD pathological factors involved in IgG’s penetration into the brain parenchyma.

In summary, our findings demonstrate the enrichment of membrane-bound IgG in microglia, and indicate its potential role in augmenting microglial function, particularly its phagocytic ability for Aβ fibrils via the IgG Fc fragment. The present study of microglia-enriched IgG not only provides insight into the role of the adaptive immune system in major neurodegenerative diseases, but also discusses the origin and transportation of brain parenchymal IgG, and potential mechanisms for its entry into the brain within the context of AD. Understanding the mechanisms involved in this process could potentially pave the way for novel AD diagnostic methodologies.

## Additional files:

***Additional Figure 1:*** *FACS of serum B cells from 6-month-old wild-type and 5×FAD mice.*

Additional Figure 1 FACS of serum B cells from 6-month-old wild-type and 5×FAD mice.
There were the FACS raw data of Figure 1J. 5×FAD: Familial Alzheimer's disease mutations; FACS: fluorescence-activated cell sorting.

***Additional Figure 2:*** *Secondary antibody against-rat IgG shows undetectable signal.*

Additional Figure 2 Secondary antibody against-rat IgG shows undetectable signal.
Scale bar: 30 μm. 5×FAD: Familial Alzheimer's disease mutations; Iba1: ionized calcium binding adaptor molecule 1; IgG: gamma-type immunoglobulin.

***Additional Figure 3:*** *Isolation of highly purified microglia for transcriptome analysis of the effect of IgG exposure on microglia.*

Additional Figure 3 Isolation of highly purified microglia for transcriptome analysis of the effect of IgG exposure on microglia.
(A) The diagram depicting microglia purification procedures using magnetic-activated cell sorting system. (B) The immunostaining assay showed a predominant proportion of the purified cells expressed microglia marker Iba1 (red, Alexa Fluor 568). Scale bar: 30 μm. (C) The flow cytometry confirmed that 99% of the purified cells expressed microglia marker CD11b. (D) The RNA-seq of purified microglia with or without 40 μg/mL IgG treatment for 24 hours (as in Figure 5 A). (E) Among the identified 12,591 genes, 1831 were differentially expressed in microglia by 40 μg/mL IgG treatment: 944 up, and 887 down. (F) The heatmap plot of differentially expressed genes between primary microglia treated with IgG (w IgG) and without IgG (w/o IgG). (G) The linear regression shows that RNA-sequencing and qPCR results are positively correlated (R^2^ = 0.9804). DAPI: 4’,6-Diamidino-2-phenylindole; Iba1: ionized calcium binding adaptor molecule 1; IgG: gamma-type immunoglobulin; PI: propidium iodide; qPCR: quantitive polymerase chain reaction; RNA-seq: RNA-sequencing.

***Additional Figure 4:*** *IgG enhances microglia phagocytic capacity for A*β *fibril.*

Additional Figure 4 IgG enhances microglia phagocytic capacity for Aβ fibril.
(A, B) The representative images showing IgG enhanced microglia’s phagocytic capacity for FITC-labeled Aβ fibril in serum-free culture medium with (B) or without (A) IgG supplement. (C) The quantification of IgG’s effect on microglia phagocytosis of FITC-labeled Aβ fibril. Data are expressed as mean ± SD, and were analyzed by two-tailed Welch’s *t*-test. (D) The flow cytometry quantification of IgG’s effect on microglia phagocytosis of FITC-labeled Aβ fibril. Aβ_f_: Amyloid-beta fibril; DAPI: 4’,6-diamidino-2-phenylindole; FITC: fluorescein isothiocyanate; Iba1: Ionized calcium binding adaptor molecule 1; IgG: gamma-type immunoglobulin.

***Additional Table 1:*** *Primers for quantitative polymerase chain reaction.*

***Additional Table 2:*** *Antibodies for immunohistochemical and immunocytochemical stainings.*

## Data Availability

*The RNA sequencing data can be accessed on https://ngdc.cncb.ac.cn/ via the accession number CRA014906.*
